# Determinants of functionality and effectiveness of community health workers: results from evaluation of ASHA program in eight Indian states

**DOI:** 10.1186/1753-6561-6-S5-O30

**Published:** 2012-09-28

**Authors:** Thiagrajan Sundararaman, Rajani Ved, Garima Gupta, M Samatha

**Affiliations:** 1 National Health Systems Resource Centre, New Delhi, India

## Introduction

In 2005, Accredited Social Health Activist (ASHA, a female community health worker), program was launched as part of the National Rural Health Mission (NRHM), a flagship program of the government of India. With 846,809 ASHAs across 31 states and union territories, the program has grown to become the most important facet of the NRHM. The ASHA is a woman selected by the community, who is trained and deployed to function in her own village to improve the health status of the community. However, despite being hailed as the face of the NRHM, several operational issues lack clarity and hinder her functions. These include clarity on her role, expected outcomes, adequacy and quality of training and support systems, and defining her future role.

## Methods

The objectives of the evaluation included (1) understanding the evolution of the program, perspectives, and experiences of key stakeholders in specific context; (2) assessing the functionality of ASHA in relation to her effectiveness in bringing health outcomes, and (3) reviewing the quality of key mechanisms that constitute the program.

The evaluation adopted a realistic approach with qualitative and quantitative inputs. Eight states were chosen purposively to yield maximum insight in divergent mechanisms, contexts and outcomes. Within each state, two districts were chosen on criteria of high and moderate performance as ascertained by the state governments. Sample size for each district included: 100 ASHAs, 600 service users, 25 Auxiliary Nurse Midwife (ANMs), 100 *Anganawadi* workers (AWW), and 100 members from *Panchayati Raj* (self-governance) institutions from 100 villages. The evaluation was designed to distinguish between functionality (defined as specific tasks carried out by ASHA) and effectiveness (defined as desired change in health behavior or improved access to health service that is measurable).

## Results

The ASHA guidelines issued in 2006 outlined three key roles of ASHA; a healthcare facilitator to facilitate access to care, a community level care provider for a limited range of services, and a health activist. The findings show that states studied adapted the guidelines to suit their interpretation of the roles of ASHA and this affected the nature of support and training provided to ASHAs.

Utilization of services provided by ASHAs shows that programmatic emphasis on care for the pregnant women have resulted in 75% of pregnant women across the states receiving services from ASHA, with some divergences. About 75% of the sampled service users received counseling on breastfeeding, but this was 60% for other aspects notably warmth and postpartum care. Service overage for children with any episode of illness in last one month was on an average 70% across states with lowest figures being reported from Bihar and Jharkhand. Evaluation shows that vast majority of ASHAs are functional, irrespective of context, although there is a wide variation in the tasks that she does. However, despite her being ‘functional’, effectiveness varies depending on systems response or skill sets. Educational qualification did not make a difference to health outcomes, but duration and content of training did.

## Discussion

We conclude that for an ASHA to be effective, all three roles are important and complementary in nature. The functionality of ASHAs in one role is clearly linked with better outcomes in other two roles. The evaluation also notes that prioritization of only the link worker function, fails to make full use of her potential for child survival and further reduces her ability to reach the marginalized communities. Comparing across states, the subjective program theory of the managers emphasized different operating mechanisms and that in turn influenced program outcomes. We recommend that, beyond provision of cash incentives, a greater support should be given to the provision of competency based training, the health rights dimension, an adequate supply of medicines, and mentoring and motivation to ASHAs.

## Competing interests

Authors declare that they have no conflict of interest.

## Funding statement

The study was funded by the Ministry of Health and Family Welfare, Government of India.

